# A 0.35-μm CMOS-MEMS Oscillator for High-Resolution Distributed Mass Detection

**DOI:** 10.3390/mi9100484

**Published:** 2018-09-22

**Authors:** Rafel Perelló-Roig, Jaume Verd, Joan Barceló, Sebastià Bota, Jaume Segura

**Affiliations:** System Electronic Group (Physics Department), Universitat de les Illes Balears, Palma 07122 (Balearic Islands), Spain; rafel.perello@uib.es (R.P.-R.); j.barcelo@uib.es (J.B.); sebastia.bota@uib.es (S.B.); jaume.segura@uib.es (J.S.)

**Keywords:** MEMS resonators, mass sensors, CMOS-MEMS, pierce oscillator

## Abstract

This paper presents the design, fabrication, and electrical characterization of an electrostatically actuated and capacitive sensed 2-MHz plate resonator structure that exhibits a predicted mass sensitivity of ~250 pg·cm^−2^·Hz^−1^. The resonator is embedded in a fully on-chip Pierce oscillator scheme, thus obtaining a quasi-digital output sensor with a short-term frequency stability of 1.2 Hz (0.63 ppm) in air conditions, corresponding to an equivalent mass noise floor as low as 300 pg·cm^−2^. The monolithic CMOS-MEMS sensor device is fabricated using a commercial 0.35-μm 2-poly-4-metal complementary metal-oxide-semiconductor (CMOS) process, thus featuring low cost, batch production, fast turnaround time, and an easy platform for prototyping distributed mass sensors with unprecedented mass resolution for this kind of devices.

## 1. Introduction and Motivation

Micro-/nanoelectromechanical systems (M/NEMS) resonators have been extensively proposed for the detection of small concentrations of analyte molecules in a gaseous solution (e.g., detection of volatile organic compounds (VOCs) through gravimetric sensing where a shift in resonance frequency is obtained in response to an added mass over the resonator). In this way, a reduction of the resonator size provides a mass sensitivity increase since the relative mass change is bigger, and in general the resonance frequency also increases. Extremely high mass sensitivity has been reported using submicrometer and nanometer scale resonators (i.e., cantilevers and CC-beams) [1,2,3,4]. Such beam-shaped resonators are the best candidates for punctual mass detection providing also an intrinsically high spatial resolution [5]. However, in applications requiring distributed mass sensing (e.g., gas detection), a relative small device area or high beam length to width ratio may represent a drawback when a subsequent resonator surface functionalization step (e.g., by ink jet, spray coat, dip cast, dip pen, or chemical deposition) is required [6,7].

In this work, we design and fabricate an alternative resonator topology based on a plate supported by four fixed-guided beams using a commercial 0.35-μm complementary metal-oxide-semiconductor (CMOS) technology. Following the fabrication technique reported in previous works, here we demonstrate the feasibility of increasing almost two orders of magnitude the resonator capture area over previous cc-beam resonators while not only preserving the final device mass resolution (per area), but enhancing it. Therefore, the predicted minimum mass change per unit area that the sensor oscillator can detect is 300 pg·cm^−2^, which is, as far as we know, the best value reported in the literature for a monolithically integrated CMOS-MEMS device.

The paper is structured as follows: Section 2 deals with the description of the oscillator device, while in Section 3 we derive an analytical model where the resonator design parameters consider both its mass sensitivity and motional resistance with enough accuracy. In Section 4 the monolithic fabrication approach is described, and the electrical characterization results are reported. Finally, a comparative analysis with the state-of-the-art and conclusions are included in Section 5.

## 2. Device Description

**Figure 1:**
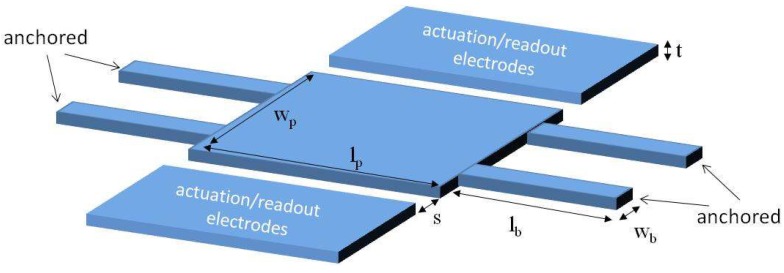
Schematic showing resonator dimension parameters.

The resonator device consists of a lateral moving plate supported by four fixed-guided beams, and two electrodes, one for electrostatic actuation, and the other for capacitive readout as illustrated in Figure 1. The top metal layer available in the commercial 0.35-μm CMOS technology is used as the physical layer to fabricate these mechanical structures [8] having an equivalent thickness (*t*) of 850 nm, approximately. The proposed resonator structure increases the capture area compared to the beam shaped resonators developed in previous works [9] meant for punctual mass sensing. Four anchored beams are included in the design to provide stability and to promote lateral vibration when excited by the electrodes. As further addressed in Section 3, the design parameters are chosen to optimize the distributed mass sensitivity while keeping a capture area large enough. The resonator plate area, or sensor capture area, is 41 μm (*l_p_*) × 10.2 μm (*w_p_*). Each beam length (*l_b_*) is 10 μm and the width (*w_b_*) is 0.8 μm. The gap between the resonator and the electrodes is determined by the minimum metal layer spacing allowed by the technology (*s* = 0.6 μm).

The adoption of a single CMOS layer is inherently superior in mass sensitivity than the option of a stack of layers, given its lower mass. However, this implies also a lower capacitive coupling between the driver and the resonator constraining the resonator motional current detection, as detailed in next section. To overcome these issues, an on-chip high-sensitivity circuit has been integrated.

**Figure 2:**
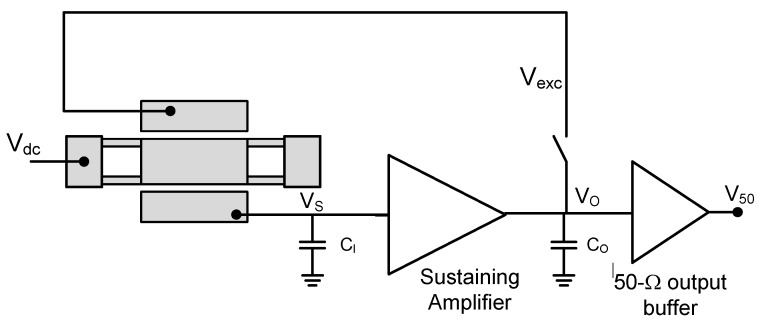
Conceptual circuit schematic of the lateral moving plate resonator into a Pierce oscillator topology.

Moreover, to obtain a feasible system-on-chip, not only the readout pre-amplifier is integrated but also a full oscillator circuit scheme based on a modified Pierce topology is included, as shown in Figure 2, obtaining a quasi-digital output signal when working in closed-loop mode. The main challenge of adapting an oscillator circuit for a CMOS-MEMS resonator is its very large equivalent motional resistance (usually in the MΩ range) that must be compensated by the sustaining amplifier to enable the self-sustaining oscillator performance. The Pierce oscillator topology is used in this case, since it is superior to a transresistance amplifier in terms of the oscillator noise figure when high transimpedance gains are used [9], like that required in this work. The reason is due to the fact that most of the gain is provided by a noiseless capacitive input element rather than a lossy resistive element. In this case, the circuit exhibits a transimpedance gain of 11 MΩ and an input-referred current noise of 87 fA·Hz^−1/2^ at 6 MHz.

In addition to the self-excited oscillator mode (closed-loop), the system has been designed to allow its operation also in open-loop mode (illustrated as a switch in Figure 2), thus enabling a way to characterize and test the proper resonator behavior.

## 3. Analytical Modelling

An approximate analytical model of the capacitive plate resonator is used in this work to estimate the resonance frequency, mass sensitivity, and electromechanical motional resistance, thus enabling a proper and simple design of the MEMS parameters. The system is conceived as a lumped mass-spring-damper model with a motion equation governing the mechanical resonator dynamics in one dimension (lateral in our case), as given by:

(1) $$\;m_{\mathrm{eff}}\ddot{x}+\gamma \dot{x}+kx=F_{\mathrm{exc}}\;$$

where $\dot{x}$ and $\ddot{x}$ represent the first and second time derivative of the position variable *x*, *m*_eff_ is the resonator effective mass, *γ* is the damping coefficient, *k* is the linear spring constant of the system, and *F*_exc_ is the net electrostatic force. The system resonance frequency is obtained as *f*_res_ *= (k/m*_eff_*)* ^1/2^.

The spring constant expression of each single fixed-guided beam is [10]:

(2) $$\;k_{b}=\frac{Ew_{b}^{3}t}{l_{b}^{3}}\;$$

where *E* is the Young modulus of the structure material (top metal layer in this case). For the plate supported by four fixed-guided beams, the equivalent spring constant is:

(3) $$\;k_{t}=4\frac{Ew_{b}^{3}t}{l_{b}^{3}}\;$$

The total dynamic resonator mass is calculated considering that the plate is completely rigid compared to the beams. Therefore, the system mass can be expressed in terms of the entire plate mass and the effective mass contribution of each guided-beam [11]:

(4) $$\;m_{\mathrm{eff}}=\rho l_{p}w_{p}t+4\times 0.37\rho l_{b}w_{b}t\;$$

Using the previous equations and assuming that the mass of the platform is larger than the one of the beams, the resonance frequency is found to be proportional to the resonator design parameters as $\sqrt{\frac{w_{b}^{3}}{l_{p}w_{p}l_{b}^{3}}}$. The resonator mass sensitivity can be expressed in terms of last parameters as:

(5) $$\;S_{m}=-\;\frac{2\times m_{\mathrm{eff}}}{f_{o}}\;$$

The negative sign indicates that an added mass over the resonator results in a decrease of its resonance frequency. For distributed mass deposition (e.g., mass sensing in a gaseous solution), the sensitivity per unit of area is defined as:

(6) $$\;S_{\frac{m}{a}}=-\;\frac{S_{m}}{A_{\mathrm{eff}}}\;$$

From the last equations, it can be easily deduced that the distributed mass sensitivity is proportional to the resonator dimensions as $l_{p}w_{p}\sqrt{\frac{l_{b}^{3}}{w_{b}^{3}}}t$.

On the other hand, from the point of view of the electrostatic transduction efficiency or capacitive readout performance, the resonator is required to exhibit the minimum possible motional resistance defined as:

(7) $$\;R_{m}=\;\frac{\sqrt{k_{t}m_{\mathrm{eff}}}}{Q\eta^{2}}\;$$

where *Q* is the resonator quality factor related to the system damping. Considering the electrode-resonator interface as a parallel plate variable capacitor, the electromechanical transduction factor *η* is given by:

(8) $$\;\eta =V_{\mathrm{dc}}\;\frac{C_{o}}{s}=\;V_{\mathrm{dc}}\;\frac{\varepsilon_{o}l_{p}t}{s^{2}}\;$$

and therefore, the motional resistance can be also related to the resonator dimensions in this case as $\sqrt{\frac{w_{b}^{3}w_{p}}{l_{b}^{3}l_{p}^{3}}}\frac{s^{4}}{t}$.

**Figure 3:**
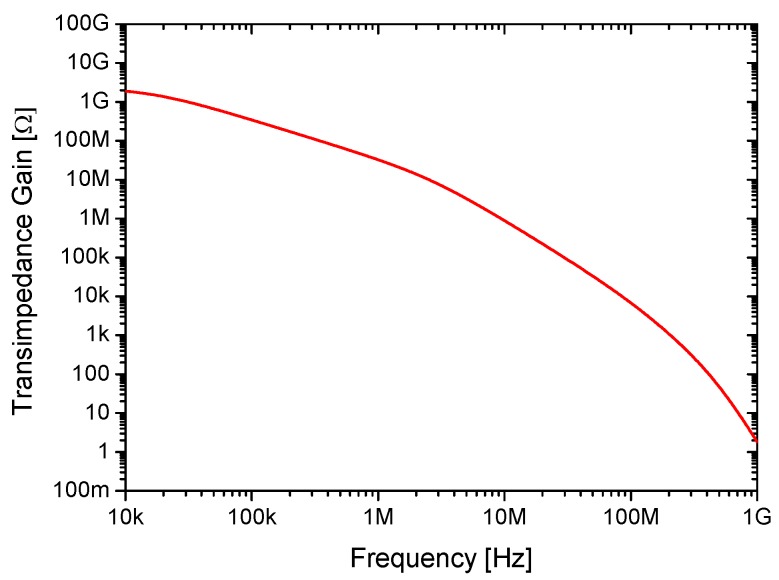
Transimpedance gain versus frequency of the sustaining amplifier circuit.

For a proper performance of the resonator in terms of mass sensitivity, but also of capacitive reading capabilities, it is required that both *S_m_* and *R_m_* resonator parameter values to be as small as possible. As deduced from the last equations, there is a trade-off between both parameters. While the mass sensitivity can be improved by decreasing the resonator mass and increasing both its capture area and resonance frequency, the motional resistance is reduced by increasing the resonator mass and lowering its resonance frequency. Such a trade-off is noticed mainly in terms of the guided-beams dimensions (*l_b_* and *w_b_*). In this sense, the resonator has been designed to exhibit the best mass sensitivity while exhibiting a motional resistance below the transimpedance gain of the CMOS sustaining circuit. Such gain must overcome the motional resistance to enable a self-sustaining oscillator operation [9]. Moreover, the frequency must be also constrained to the CMOS amplifier specifications. Figure 3 shows the dependency of the circuit transimpedance gain used in this work with the operating frequency. Since in the Pierce configuration the sensed current is integrated through the capacitance at the sustaining amplifier input (*C_i_* in Figure 2), the amplifier transimpedance gain decreases with frequency.

Therefore, the beam width (*w_b_*) is chosen as the minimum available by technology since a wider beam highly increases both the frequency and the motional resistance. The platform width and length (*w_p_* and *l_p_*) are chosen to fulfill the capture area requirements, with the length larger than the width, to avoid increasing the motional resistance unnecessarily. Thus, the parameter available for the design flow is the beam length (*l_b_*). Finally, we also set the gap between the resonator and the electrode (*s*) as the minimum available by the technology to minimize the motional resistance as well. In any case, large motional resistance values are still obtained since the use of a single top metal layer with a relatively small thickness and a relatively large minimum distance as indicated in Section 2.

This analytical model has been used to design the resonator parameters according to the values indicated in Section 2. Additionally, we ran extensive finite element modeling (FEM) simulations using COMSOL Multiphysics to validate the model parameters prior to fabrication. Comparison of the accurate finite element modeling (FEM) values to the analytically derived parameters revealed that a high accuracy of such a simple model is good enough to design the resonator parameters and predict its main performance as a sensor device. Considering that the spring constant may usually vary by 10%–20% due to process variations in conventional MEMS fabrication techniques [10], the results detailed in Table 1 are absolutely acceptable.

## 4. Fabrication and Electrical Characterization

**Figure 4:**
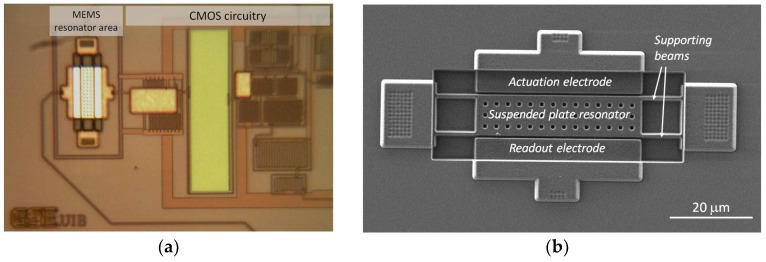
Fabricated device in a complementary metal-oxide-semiconductor (CMOS) 0.35-μm commercial technology constituted by a plate resonator integrated monolithically with CMOS circuitry: (**a**) Optical image of the overall CMOS-MEMS oscillator circuit; (**b**) SEM image of the metal suspended plate resonator.

The MEMS device was completely defined along the commercial 0.35-μm CMOS process by using the top metal layer available in the technology used [8]. The silicon oxide underneath the resonator was used as the sacrificial layer that was removed after the standard CMOS process by means of a one-step maskless wet etching. This construction scheme provides an easy monolithic integration of the mechanical resonator with the CMOS circuitry (see Figure 4a). In this case, various openings were defined in the plate structure to ease the wet etching process (Figure 4b).

**Figure 5:**
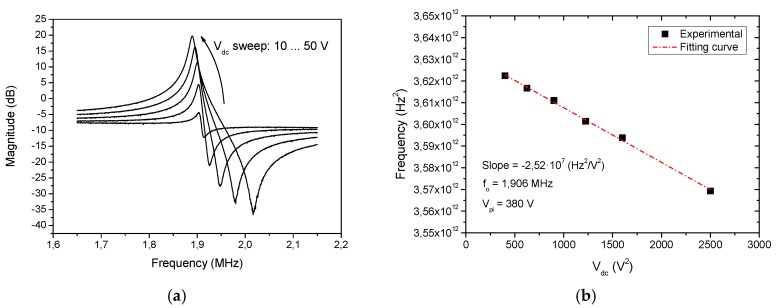
Electrical characterization of the suspended plate resonator with on-chip 0.35-μm CMOS readout circuit in open-loop configuration: (**a**) Measured frequency response (magnitude) for different resonator bias voltages (*V*_dc_) in air conditions; (**b**) Plot of the resonance frequency dependency versus the applied bias voltage.

The CMOS-MEMS device has been characterized both in open-loop and closed-loop mode in air conditions (atmospheric pressure and ambient temperature). Open-loop measurements have been performed at a low excitation power (−30 dBm) to operate in the resonator linear region, enabling the extraction of the linear electromechanical parameters. Figure 5 shows the measured electromechanical system transmission response magnitude obtained for various resonator bias voltages *V*_dc_. The resonance frequency of the MEMS resonator is around 1.9 MHz and its quality factor is *Q* = 176. On the other hand, it is noticed that the spring softening effect (the decrease of resonant frequency when the bias voltage increase) is not marked in contrast to the one exhibited by clamped-free resonators. From such behavior (Figure 5b), the pull-in voltage of the device is predicted to be as high as 380 V.

**Figure 6:**
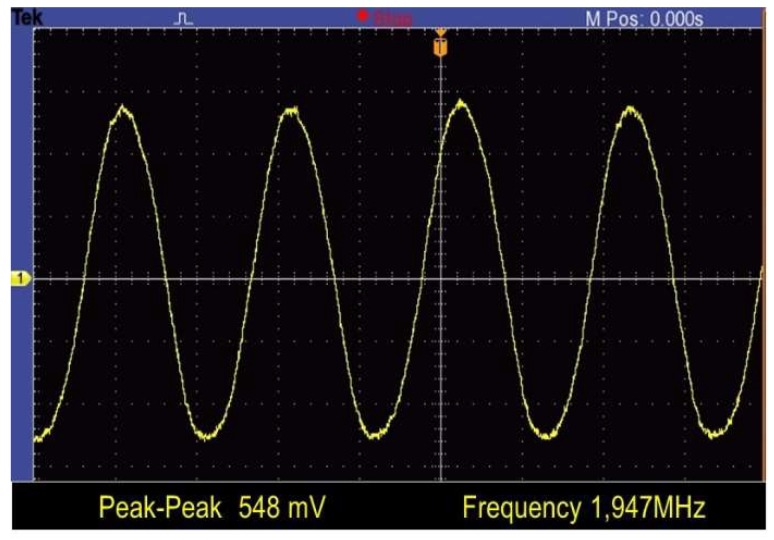
Time-domain oscillator output signal measured for *V*_dc_ = 27 V.

**Figure 7:**
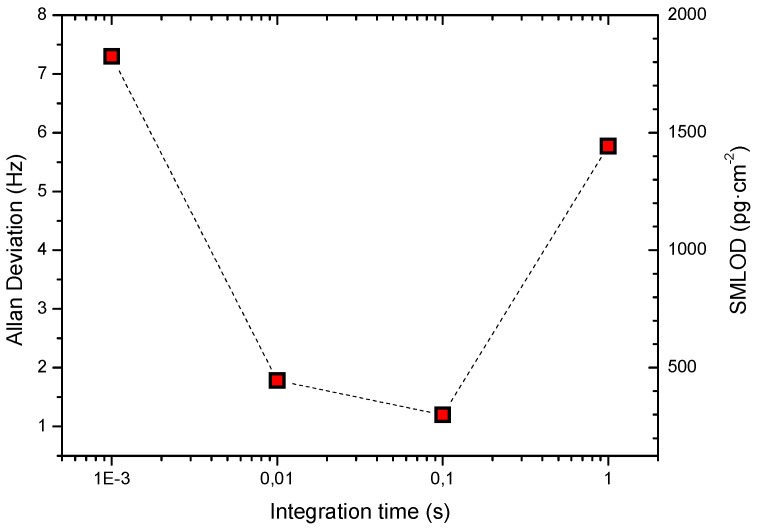
Allan deviation as a function of integration time measured in air conditions (atmospheric pressure and ambient temperature). The corresponding surface mass limit of detection (SMLOD) for each integration time is indicated in the right y-axis.

The oscillator output signal behavior, when working in self-excited mode (closed-loop measurements), is depicted in Figure 6. The oscillator works properly with biasing voltages over 25 V. For a *V*_dc_ = 27 V, the measured oscillator generates a 1.947 MHz signal with a peak-to-peak amplitude beyond 500 mV. In resonant sensing, where the measurement is performed by tracking the MEMS resonance frequency variation, the Allan deviation parameter of the oscillator frequency becomes a key parameter widely used to assess the short-term stability of oscillators and to predict, in this case, the short-term resolution of the device (e.g., mass resolution). In this work, a precise frequency counter (CNT-90, Pendulum, Banino, Poland) has been used to measure the Allan deviation of the CMOS-MEMS oscillator in a range from 1 ms to 1 s integration times (Figure 7). The integration time is equivalent, in the time domain, to the measurement bandwidth in the frequency domain used in other instruments like spectrum analyzers. 

The corresponding surface mass limit of detection (SMLOD) is also provided in Figure 7. This parameter accounts for the minimum mass change per unit area that the CMOS-MEMS device can detect. It has been computed from the predicted mass sensitivity times of the measured Allan deviation value. The minimum Allan deviation value is obtained for an integration time of 100 ms being as low as 1.2 Hz or 0.63 ppm.

## 5. Comparative and Conclusions

The plate resonator reported in this work has a capture area ~40 times higher than featured by the cc-beam resonator used in previous works [12], and is thus more suitable for distributed mass sensing applications as introduced in Section 1. On the other hand, its higher mass and lower resonant frequency results in a predicted mass sensitivity per unit area to be ~7 times worse (250 pg·cm^−2^·Hz^−1^). However, the better frequency stability of the device results in a predicted surface mass limit of detection (SMLOD) to be as low as 300 pg·cm^−2^, and is thus better than obtained for the cc-beam resonator in air conditions. In addition, the plate resonator operates at a lower bias voltage. In our opinion, the better oscillator frequency stability is related to a higher mechanical linearity and quality factor that exhibits this type of MEMS structure compared to the single cc-beam previously used in [12]. Being an open topic, we are currently investigating in this direction to corroborate such assumption.

The predicted SMLOD value and the expected mass sensitivity per unit area obtained in this work is also the best compared to the state-of-the-art for monolithic CMOS-MEMS oscillators (with on-chip feedback circuitry) operating in air conditions, as shown in Table 2. Only a few works addressing CMOS compatible MEMS mass sensors report smaller SMLOD values [13,14]. However, the better frequency stability in [14] is obtained thanks to the use of external off-chip feedback circuitry based on a phase locked loop (PLL). In [13], they also used an off-chip PLL, and the better mass sensitivity is inherent to the use of a nano-scale cantilever structure having a 10^2^ times smaller capture area. On the other hand, comparison with non-CMOS/MEMS resonators reveals that only some works based on piezoelectric transduction and fabricated using specific nanofabrication techniques exhibit better performance in terms of SMLOD (e.g., in References [15,16]).

As a main conclusion, in this work we have experimentally demonstrated the feasibility of constructing a fully integrated and fully standard CMOS fabrication process compatible MEMS-based oscillator with a surface beyond 60 times of single cc-beams with a superior SMLOD in air conditions. Given its large area and high mass sensitivity per unit area, this device is a perfect candidate for applications requiring selective compound detection in gas environments where functionalization steps are mandatory. The higher capture area ensure proper adherence of the subsequent chemical species, thus avoiding the contour-dominated effect intrinsic disadvantages of narrow cc-beams.

Finally, the full CMOS/MEMS integration of the mechanical and electronic circuitry and the quasi-digital output provided by the oscillator makes this solution a perfect candidate for low cost system-on-chip or Lab-on-chip applications.

## Figures and Tables

**Table 1:** Main resonator parameters comparison obtained from finite element modeling (FEM) simulations and using the approximated analytical solution. A mass density *ρ* = 3000 kg/m^3^ and a Young’s modulus *E* = 131 GPa have been assumed for the complementary metal-oxide-semiconductor (CMOS) top metal layer to compute the parameters.

Parameter	FEM	Analytical	Error (%)
Resonance frequency, *f_o_* (MHz)	2.124	2.296	−7.5
Linear stiffness, *k* (N/m)	199.8	228	−12.3
Mass sensitivity, $S_{\frac{m}{a}}$ (pg·cm^−^^2^·Hz^−1^）	246.8	228.5	8.0

**Table 2:** State-of-the-art of CMOS-MEMS resonators as distributed mass sensors operating in air conditions.

Ref.	Sensitivity (pg·cm^−2^·Hz^−1^)	Capture Area (cm^2^)	Frequency Stability (Hz)	SMLOD (pg·cm^−2^)	Integrability
This work	250 *	4.2 × 10^−6^	1.2	300 *	On-chip oscillator
[12]	34 *	1.1 × 10^−7^	15	510 *	On-chip oscillator
[13]	0.10 *	1.2 × 10^−9^	20	2 *	Off-chip PLL
[14]	2300	2.2 × 10^−4^	0.08	190	Off-chip PLL
[17]	61,000	2.3 × 10^−4^	0.03	1800	On-chip oscillator
[18]	240,000	1 × 10^−4^	~1	240,000	On-chip oscillator

* Predicted value.
